# An apparent quandary: adoption of polygenics and gene panels for personalised breast cancer risk stratification

**DOI:** 10.1038/s44276-023-00014-w

**Published:** 2023-09-18

**Authors:** Jerry S. Lanchbury, Holly J. Pederson

**Affiliations:** 1 Independent Researcher Salt Lake City, UT, USA; 2 Medical Breast Services, Cleveland Clinic, Cleveland, OH USA; 3 Department of Medicine, Cleveland Clinic Lerner College of Medicine, Cleveland, OH USA

## Abstract

Over the past 30 years, genetic and epidemiological advances have revolutionised the prediction of breast cancer risk in women with significant family history. By screening these women for high- and intermediate-risk pathogenic variants and by interrogating their genomes for multiple lower-risk single-nucleotide polymorphisms (SNPs), we can provide individually tailored risk profiles in carriers of Mendelian breast cancer risk variants and in non-carriers, but clinical implementation of this approach is suboptimal. Risk mitigation may involve enhanced surveillance, preventive medications or risk-reducing surgery but barriers exist to the adoption of polygenic risk score (PRS)-based models in the clinic. PRS development has suffered from both systematic biases resulting from development and validation in those of European ancestry and from the consequences of unanticipated evolutionary differences particularly with regard to those of African ancestry. PRS approaches which take into account underlying genetic diversity offer a practical solution to the misapplication of European-derived PRS to other population groups including women of multiple ancestries. All ancestry PRS technology offers net benefit regardless of potency differences. While the new science of polygenics has surged ahead and its stratification insights have been incorporated into risk modelling, training of providers and genetic counsellors lags far behind and an educational revolution is also necessary to provide optimal patient care.

## History

Human genetic advances unpacking the molecular basis of breast cancer risk have revolutionised precision medicine in the past 30 years. Development of the field of human genetics has been dominated by an understanding of Mendelian science. Generations of medical students have studied dominant and recessive inheritance [1]. The dominant segregation of breast cancer was first recognised by Eldon Gardner’s study of Utah cancer families in the 1940s. Mapping *BRCA1* and *BRCA2* genes using genome-wide polymorphic DNA markers confirmed the existence of cancer predisposition genes. These tumour suppressor genes were first localised to chromosomes 17 [2] and 13 [3]. Mark Skolnick and team cloned and sequenced *BRCA1* and described the first pathogenic variants (PVs) in 1995 [4]. (Pathogenic variants are genetic alterations that increase an individual’s susceptibility or predisposition to a certain disease or disorder. In cancer genetics, a “likely pathogenic” variant and a “pathogenic” variant are both considered to be positive results.) *BRCA2* was characterised in 1995 by groups led by Skolnick and Michael Stratton [5] Tens of thousands of distinct PVs have been characterised in these two genes and since 1996 an industry has arisen to support testing. Naturally, the medical community has embraced these exciting advances.

## Clinical utility of *BRCA* testing

We can offer targeted treatment of cancers in *BRCA* carriers utilising tumour vulnerability consequent on DNA repair defects [6, 7]. Next-generation sequencing has led to identification of a number of other genes associated with an increased risk for breast cancer [8] and this technology has resulted in more widespread multi-gene panel testing (MGPT) to inform preventive strategies in affected families. Multiple studies of familial and sporadic breast cancer cases have established *BRCA1*, *BRCA2*, *PALB2*, *TP53*, *PTEN*, *CDH1*, *STK11*, *CHEK2*, *ATM*, *NF1* and *BARD1* as the primary genes of interest for breast cancer risk [9–13]. Evidence is less secure for *RAD51C* and *RAD51D* due to rarity, strength of effect, specific phenotypic association or a combination of factors.

Both short-term (5 and 10 years) and estimated lifetime risks are used in risk management counselling. Certainly, knowing one’s estimated lifetime risk for a disease is helpful in making risk management choices, and guideline-driven thresholds for germline testing based on the pre-test probability of identifying an abnormality are in place to target the testing of individuals at the highest risk [14]. Knowledge of shorter-term risks may reassure younger childbearing women that their absolute estimated risks during that period are relatively low; similarly, an older woman with a *BRCA2* PV has both lower short- and long-term risks—she may make very different decisions than her younger daughter. High-risk women are offered enhanced surveillance (adding annual contrast-enhanced breast magnetic resonance imaging), consideration of risk-reducing medications or even discussion of risk-reducing surgeries [15, 16]. Depending on her level of risk and estimated age at onset of disease, guidelines aid in informing individual patient risk management plans [14, 15]. For instance, patients harbouring PVs in *BRCA1* and *BRCA2* are counselled similarly with regard to short- and long-term risks [17], the anticipated age at onset of breast cancer [17] and recommended risk management strategies [14, 15] as well as opportunities for reproductive assistance; however, patients with *BRCA1* PVs must appreciate their predisposition to early-onset triple-negative breast cancer [18] in making personal risk management decisions. Suggested “thresholds of risk” may help inform the appropriate ordering of and reimbursement for MRI screening, and this threshold may vary regionally. Thresholds of risk have also been suggested for consideration of preventive medication [15, 19], but patient preference and comorbidities must be considered. Discussion around risk-reducing mastectomy is generally recommended for those at the highest levels of risk, and is a very personal decision, often influenced by cultural mores. Early identification of those at hereditary risk allows for optimisation of risk reduction strategies in affected individuals.

Adoption of breast cancer risk estimation, however, has been slow and fraught with unnecessary impediments. Initially, concern existed over whether women were competent to receive and act upon their *BRCA1* and *BRCA2* mutation status (and later the results of MGPT testing [20]); many women have since embraced this robust testing technology, empowering them to make informed, proactive risk-management choices; patients are interested in knowing their genetic information. A multicentre prospective diverse cohort examined the diagnostic yield and patient experience of MGPT for hereditary cancer risk. 80% of patients wanted to know all results, “including findings that doctors do not fully understand” [21]. Patients also independently seek genetic information, as evidenced by the often misinterpreted results obtained through the booming direct-to-consumer market.

## Adoption of *BRCA* and panel testing

Max Planck famously stated, “A new scientific truth does not triumph by convincing its opponents and making them see the light, but rather because its opponents eventually die, and a new generation grows up that is familiar with it”. At the time of writing, at least 5 million women in the United States will have experienced a comprehensive screen for PVs/likely pathogenic variants (LPVs) in *BRCA1* and *BRCA2* yielding around 100,000 carriers. According to the United States 2020 census, the US adult population grew 10% since 2010, reflecting aging baby boomers [22]. Assuming women to be half of the 258,343,281 adults, and assuming a very conservative population mutation prevalence on MGPT of 1.63% [10], screening every woman in the US has the potential to identify over 2 million gene carriers in one year alone. Assuming a 2.5%/year risk of breast cancer annually [17] this identification has the potential to prevent up to 525,000 cases over a 10-year period and conservatively, $40 billion in healthcare costs. In the United Kingdom, *BRCA* genetic testing is rationed to individuals with a pre-test probability of 10% or greater per NICE guidelines that can be accessed at https://www.nice.org.uk/guidance/cg164/ifp/chapter/genetic-counselling-and-genetic-testing.

The concern for substratification of breast cancer risk and identification of those at hereditary risk is shared by both patients and providers. Ideally, a woman would know her germline predisposition *before* she develops cancer. Mary-Claire King, credited with discovery of the first DNA linkage to breast cancer, asserted in 2014 on winning the Lasker Award for Science, that *all* women consider *BRCA* testing prior to the age of 30 years [23]. The American College of Radiology in 2023 stated that “All women should undergo risk assessment no later than age 25, especially Black women and women of Ashkenazi heritage, so that those at higher-than-average risk can be identified and appropriate screening initiated”. [24] In two large community series, one in four patients met criteria for genetic testing [25, 26]. From a large population-based case–control study by Couch, pathogenic variants in 12 established breast cancer-predisposition genes were detected in 5.03% of cases and in 1.63% of controls [12]. Further, even when identified and followed closely, these women face daunting risks. Warner et al. observed 489 women with pathogenic variants in *BRCA1* and *BRCA2* involved in an MRI screening programme for an average of 13 years. Of those diagnosed, 26% were diagnosed at Stage 2 or higher and 52.2% required chemotherapy [27]. Early identification, risk management counselling and close follow-up are critical for this group of patients to limit disease burden and morbidity. We believe that ultimately universal genetic and genomic screening will be offered to women to substratify their breast cancer risk.

## Comprehensive breast cancer lifetime risk estimates require germline sequencing, SNP profiling and evaluation of traditional risk factors

Breast cancer risk is comprised of genetic and non-genetic factors. Non-genetic factors include reproductive factors and possibly contraceptive choices, body mass index, extended use of combined post-menopausal hormones, alcohol, physical inactivity, benign atypical lesions on breast biopsy, therapeutic chest irradiation and breast density. Genetically, there are three classes of known breast cancer susceptibility alleles stratified by frequency and degree of risk conferred: very rare highly penetrant alleles with a relative risk (RR) of >4, rare moderately penetrant alleles (RR of 2–4), and common low-penetrance alleles (RR of <2) [28]. This latter class is mostly composed of SNPs which are weighted and combined into summary risk metrics called polygenic risk scores (PRSs). The medical community has so far failed to embrace the application of models incorporating PRSs. The reasons are multifaceted; some barriers are similar to those encountered in germline testing, while others are unique to the new science of polygenics. The same controversies that exist over more widespread germline testing will surely apply to PRS. Third-party payer reimbursement is largely governed by eligibility guidelines set forth by NCCN [16] which currently states that “use (of PRS) is recommended (only) in the context of a clinical trial, ideally including more diverse populations” [16]. In families with negative germline testing, or in whom a variant of unknown significance (VUS) is found (a VUS is a change in the DNA for which the clinical impact is unclear and is not used clinically to inform patient or family risk management), individuals are currently managed based on risk models [15]. Further risk stratification is needed in these individuals, and one next step is in the determination of PRS and integration with risk models such as Tyrer-Cuzick and CanRisk [29]. It is also critical that genetic counsellors understand and embrace this technology as they translate the science of genetic and genomic technology into a conversation a patient can process to make informed decisions about their risks and options.

Evidence continues to accumulate exploring clinical utility in the areas of ongoing risk substratification, ancestry differences, risk-based screening, and even the prediction of more aggressive breast tumours. A large case: control study published in 2022 examined the additive impact of the 313-variant PRS in unaffected BRCA-negative women. Inclusion of the PRS led to substantially different recommendations in 34% of the patients than would have been informed by family history alone [30]. Risk-based screening implementation including use of the PRS would likely be cost-effective and would minimise the “harms” associated with screening mammography, while providing enhanced surveillance to those identified as being at increased risk [31]. In a large modelling analysis examining use of the PRS to identify high risk patients, the PRS defined a high-risk quintile (20%) of the population which was expected to capture 37% of breast cancer cases [32]. In a survey of 227 people accessing polygenic scores on-line, however, scores were sometimes misunderstood and caused distress [33]. Knowledge about and careful, consistent communication of this risk information will be the keys to successful implementation. McCarthy et al. found that the 313 SNP breast cancer PRS was independently and significantly associated with both the diagnosis of breast cancer, and poor prognosis cancers, suggesting that this tool may inform supplemental imaging choices [34]. Shieh et al. also explored a PRS for breast cancer aggressiveness, using an integrated analysis of germline SNP and tumour gene expression data to construct a PRS associated with aggressive tumour biology and worse survival [35]. The Breast Cancer Association Consortium (BCAC) and MINDACT Collaborators evaluated the association of the 313 SNP PRS with clinicopathologic characteristics and survival following breast cancer demonstrating that this PRS may be prognostic [36]. Olipade et al. observed significant differences in mutations and gene expression between patients with genetically determined African and European ancestries which may guide future treatment strategies in diverse populations [37].

Our understanding of the polygenic nature of breast cancer risk has developed over the last fifteen years; however, medical training and continuing education curricula are lacking in genetic content. The first five single-nucleotide markers (SNPs) associated with breast cancer were discovered by a genome wide association study (GWAS) in 2007 comparing 4300 cases and 4300 controls [28]. Much larger-scale GWAS mainly in women of European descent subsequently have identified hundreds of breast cancer-associated SNPs [38–41]. In 2017, an early study examined the ability of an 18-SNP PRS to substratify breast cancer risk in a familial screening clinic [42]. The highest odds ratio per standard deviation (1.7) achieved for breast cancer used a PRS of around one million SNPs in 2020 [43]. Valid PRSs exist with intermediate numbers of SNPs.

Certain questions must be asked. Does the PRS model achieve adequate discrimination across the spectrum (the ability to identify high-risk and lower-risk individuals)? Mavaddat et al. in 2015 showed that the PRS improved risk stratification in women both with and without family history [28]. Extensive validation work is underway by Hughes, Mavaddat and others, with numerous recent publications in this area [44, 45]. The second question is how well the model is calibrated for a given population (comparing observed versus expected risk values.) Ideally, both the development and validation cohorts are prescreened for highly penetrant and moderately penetrant PVs/LPVs. These patients would initially be excluded from the analysis but could separately be further substratified with PRS [46–48]. Further studies continue to refine risk estimation with mathematical models utilising traditional risk factors [49] and incorporating the PRS such as work published by Hughes et al. [50], Choudhary et al. [51] and others.

Another question is how health care providers will responsibly communicate this complex information to patients that will ultimately improve breast cancer risk assessment, early detection and prevention. Similar concerns were raised with the advent of MGPT informing studies further assessing penetrance, uptake of screening and risk-reducing strategies and assessing access, anxiety and cost-effectiveness [52].

## Powerful PRS-based risk models are now available for women of all ancestries

Two PRS models have been extensively and rigorously validated in cross-sectional populations of several hundreds of thousands of women of self-reported European ancestry showing excellent discrimination and calibration (the 313- and the 86-SNP PRS) and may be considered suitable for implementation in both risk substratification and informing risk management decision-making [44–46, 50, 52–54]. However, this implementation, critical for healthcare, suffers from a number of deficiencies and biases. The first is that the SNP panels were initially developed and validated in European populations. However, at least two high level differences exist between the genetics of African and European (and most Asian, Native American and Hispanic) populations. Africa, being the cradle of humanity, retains an historical depth of genetic and genomic diversity found nowhere else on the planet. As successive waves of early humans left Africa to populate other areas, that gene pool was repeatedly sampled and replaced outside Africa. The out-of-Africa founder population may have been as small as 1000 individuals [55], exacerbating the lack of genetic diversity. Additional discovery biases potentially exist. In all-comers of European descent, ~85% of patients’ tumours test positive for oestrogen receptor (ER) while in descendants from Africa, the figure is reduced with a higher incidence of triple negative breast cancer (TNBC). In one study conducted in the UK, 22% of Black women had TNBC as compared to 15% of White women [56]. The risk of TNBC may even be disproportionately higher in younger Black women [57]. A consequence of this is that both the discovery and validation power for ER-positive breast cancer in African groups will be compromised using European-based PRSs. The technical and historical biases outlined above indicate that an already ancestrally biased SNP panel achieves maximum discovery power in a European-derived population and will be much less effective in those from Africa.

Despite limitations, most published breast cancer PRS studies have so far collected and used self-reported ancestry. However, SNP frequencies differ by ancestry and most current PRS are not properly *calibrated* for non-Europeans, often over-estimating risk. Further, linkage disequilibrium (LD) between SNPs and presumed functional elements differs by ancestry. (LD occurs when alleles in a given population are found together more frequently than one would expect.) Further, current PRS have weaker *discrimination* for non-Europeans. In a recent study we used self-reported ancestry for African-American (AA), European, East Asian and Hispanic women to derive group-specific SNP allele frequencies and allele-specific risk weights [58]. An independent group was then evaluated for a selected panel of ancestry informative markers and a model was constructed that delivered PRS-based breast cancer risk values for a 149-SNP panel based on a woman’s genetic ancestry. The methodology delivered effective discrimination and was well calibrated in the major ancestral groups. Several PRS have been developed for self-reported African, East Asian and Hispanic populations which show significant discrimination [59–63]. Huo et al. have argued that the African PRS is ready for clinical implementation. Tschiaba et al. have presented an ancestrally adjusted PRS designed for use in multiple ancestries [64]. However, we are unaware of a study beyond Hughes et al. that has explicitly shown appropriate calibration of ancestry-specific PRS. Molecular profiling of a real-world cohort published recently by Olopade et al. showed actionable tumour biology differences between patients of European ancestry and those of African ancestry [37].

For reasons outlined above, the predictive power of the Hughes PRS-based largely on European ancestry discovery content is most effective in self-reported Europeans, and less effective in self-reported AAs. It importantly includes the protective SNP found in Native American and Hispanic populations [58]. Utilising the PRS in a woman whose ancestry is genetically determined and is calibrated for the (weighted) differences in allelic frequencies seen in women of different ancestries, we saw significant and meaningful discrimination in each subcohort. For White or Ashkenazi individuals, the OR per SD was 1.45, for Asians 1.45, for Hispanics 1.46 and for Black/African women 1.23, and overestimation of risk was no longer seen [58]. It has been argued that given the reduced risk discrimination in AAs, PRS should not be used clinically until equivalent performance can be guaranteed between ancestral groups. This is an unfair standard from multiple perspectives. First, net benefit can be delivered to all patients even if the degree of benefit differs between ancestral groups. In the field of risk estimation, it is assumed that with additional data, the accuracy of the models will improve, particularly in underrepresented populations. This has not previously halted incorporation into clinical practice. It is clear that significant investment should be made into the discovery of polygenic risk markers in African and other populations. By analogy, in early *BRCA* testing, variant of uncertain significance (VUS) rates were higher in African descendants than in Europeans since there were fewer data in AA. This disparity was not used to deny testing to any or all groups since every group received net benefit. Denial of the benefits of PRS testing to less studied groups with more complex evolutionary histories means that all women will suffer from preventable breast cancers while waiting perhaps decades for tools with “equitable” performance. These limitations did not stop risk estimation with the Gail model before data from the Black Women’s Health study were incorporated. In fact, disclaimers persist on the NCI online version regarding validation in non-White populations, with the caveat, “further studies are needed to refine and validate these models”. [65].

Clinically, several PRS are well validated with a wealth of cross-sectional and some prospective data. GENre, for example, examined the effect of the addition of a 77-SNP PRS to traditional risk modelling in patients at elevated risk for breast cancer making decisions about preventive therapy. Increased risk incorporating PRS was associated with greater intent to take endocrine therapy (*P* < 0.001) [66]. Using a European ancestry-derived PRS in non-Europeans leads to a likely inflation of risk estimation [58, 67]. The 149-SNP all-ancestry PRS is ready for clinical application for patients with high-risk family history. It has been argued that long-term prospective studies should be conducted before adoption of PRS. Widespread adoption of screening for the 13 Mendelian breast cancer genes did not require 10-year prospective clinical trials. Further, there is already overwhelming statistical evidence of our ability to identify high-risk women through PRS-based models calibrated for their ancestral genetics. In our view, it is already unethical to ignore this information when planning and approving research studies. An obvious reference point exists. In the early days of the development of the 21-gene reverse transcriptase polymerase chain reaction-based breast cancer molecular signature, it became obvious that the high-risk group who had up to a 28% improvement in 10-year distant disease-free recurrence with chemotherapy [68] was reliably identified by the tool and consequently this group was not randomised with respect to chemotherapy in the massive and lengthy TAILORx trial [69]. It would have been unethical to do so. Since every carefully developed PRS has been well validated in adequately powered cross-sectional and longitudinal studies, the same standards should apply.

There are two distinct areas of clinical utility that must be considered in validation. The first is in risk stratification, both in carriers of PVs in breast cancer predisposition genes and in non-carriers. The second is prospective validation of the clinical utility of the tool, its applicability to enhanced surveillance, use of preventive agents and even choices regarding risk-reducing operations. We have adequate data in risk stratification which will only improve with time, and studies of clinical validation will continue to be planned. The polygenic risk score is one of many data points, amongst them family history, age at diagnosis of affected relatives and germline genetic testing results from an affected family member.

## The scientific validity of PRS-based models for breast cancer risk has arrived in advance of the education necessary for implementation

To clinically implement a PRS, education must first be the focus, both among patients and providers to clarify potential applications. The already overburdened genetic counsellor will now be tasked with counselling about an entirely new technology and its implications. Genetic risk must be combined with multiple other traditional risk factors in multi-generational models focusing on family history such as the Tyrer-Cuzick or the CanRisk model. Time, expertise and clinic workflow limit the practising primary care physician in basic carrier identification, much less complex risk modelling, incorporation of the PRS and communication of risk information. A survey of United States genetic counsellors (GCs) from October 2019 to January 2020 aimed to understand current practices with breast cancer PRS, to determine the impact of PRS on patient management and to anticipate future practices. At that time, of the GCs that had not yet ordered the test, 90% felt that they would be ordering it in the future. Reasons for not ordering the test at this time included lack of clinical guidelines, insufficient evidence of clinical utility and “lack of availability” for patients of non-European ancestry [70].

The growing accessibility of genetic and genomic information has the potential to transform oncology in a truly personalised way. Care must be taken, however, to attend to the education of the provider, explaining limitations and appropriate actionability to avoid undertreatment or overtreatment. We assert that breast cancer PRS should be implemented in the clinical care of high risk patients, but to do so responsibly will require a concerted effort in education. A large population-based sample of patients with breast cancer diagnosed in 2014 and 2015 (and their providers) was surveyed demonstrating important gaps in incorporating germline genetic testing into treatment decision-making for early-stage breast cancer. Many surgeons, for example, managed patients with VUS the same as patients with P/LP variants in *BRCA1* and *BRCA2* [71], while approximately 80% of VUS eventually are classified as benign [72].

Cancer genetic and genomic testing is revolutionising precision medicine, adding complexity to clinical communication. Knowledge among both patients and providers is sadly limited leading to confusion and misinterpretation and there is very little research in underserved populations [73]. There is great need for improvement in health literacy about cancer genetic testing in general, and now the impact of polygenics. Primary care providers interpret results for patients and help them make subsequent decisions about their care. The polygenic risk score must move forward into clinical practice, with anticipated modifications as further data become available. The level of evidence being required of PRS far exceeds that demanded of mutations over last 25 years. Mendelian mutations and PRS act in concert in a complementary way. PRS represents a powerful and relevant tool for the management of families that exhibit breast cancer “clustering” and beyond, to the population, and can substratify risk within families who do carry PVs in highly penetrant or moderately penetrant genes [41]. This approach represents an ongoing multi-disciplinary collaboration to optimise precision breast cancer risk estimation and risk management. Shared decision-making discussions are key, particularly with women whose genomic data are currently under-represented.

Understanding polygenic risk in complex human diseases likely qualifies as a scientific revolution. For the last two decades most basic discovery advances in human genetics have led to rapid adoption within clinical settings. The clinical adoption of powerful and robust risk models that include polygenic components is unfortunately proceeding very slowly. Guidelines for conducting and interpreting genetic and genomic testing results will be critical going forward to ensure ethical and safe care delivery regarding both risk estimation and the management of risk.
